# Atypical femoral fracture and jaw osteonecrosis under high doses of denosumab, healed with the aid of low dose of denosumab: a case report

**DOI:** 10.3389/fendo.2026.1761311

**Published:** 2026-02-24

**Authors:** Hanene Lassoued, Olivier Lamy, Cyril Francioli, Elena Gonzalez Rodriguez

**Affiliations:** 1 Centre Hospitalier Universitaire Vaudois (CHUV), Lausanne, Switzerland; 2 Bone Unit, Lausanne University Hospital, University of Lausanne, Lausanne, Switzerland; 3 Universite de Lausanne, Lausanne, Switzerland; 4 Department of Maxillofacial Surgery, Lausanne Hospital, CHUV, Lausanne, Switzerland

**Keywords:** atypical femor fracture, denosumab (anti-RANKL antibody), jaw osteonecrosis, low-dose, treatment

## Abstract

Atypical femoral fractures (AFF) and osteonecrosis of the jaw (ONJ) are rare but serious complications associated with long-term use of antiresorptive therapies such as denosumab. Denosumab discontinuation to allow for bone healing is not possible because of the high risk of spontaneous multiple vertebral fractures. We present a case of concurrent AFF and ONJ in a patient previously treated with denosumab 120mg monthly. The patient was managed with a low-dose denosumab regimen tailored according to bone turnover marker monitoring, resulting in successful bone union and resolution of ONJ.

## Observation

We report the case of a 56-year-old woman (written consent was obtained for publication) with a history of breast cancer (pT1c (1.1 cm) pN1mi (1mi/22), cM1 and vertebral metastases, treated with aromatase-inhibitor, radiotherapy and monthly denosumab (120 mg) since 2016.

**Figure 1:**
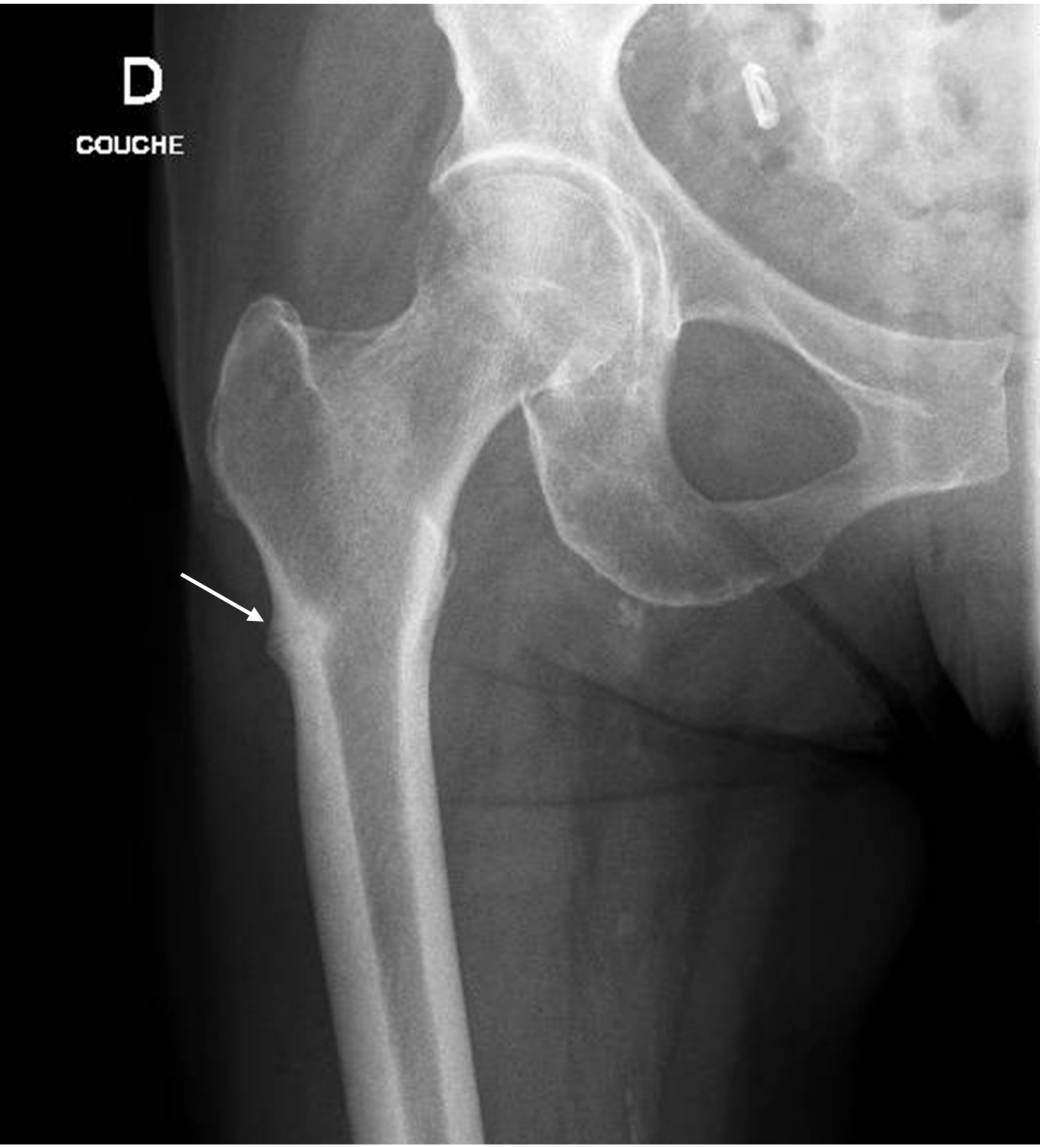
Atypical femur fracture, the arrow showing the radiolucent line on the lateral femur cortex.

In July 2022, she presented with spontaneous pain in her right femur, without any history of trauma, fall, or excessive physical effort. Radiographs revealed a transverse radiolucent line on the lateral cortex of the femoral shaft, consistent with an atypical femoral fracture (AFF) (**Figure 1**). Weight bearing based on pain tolerance was advised.

**Figure 2:**
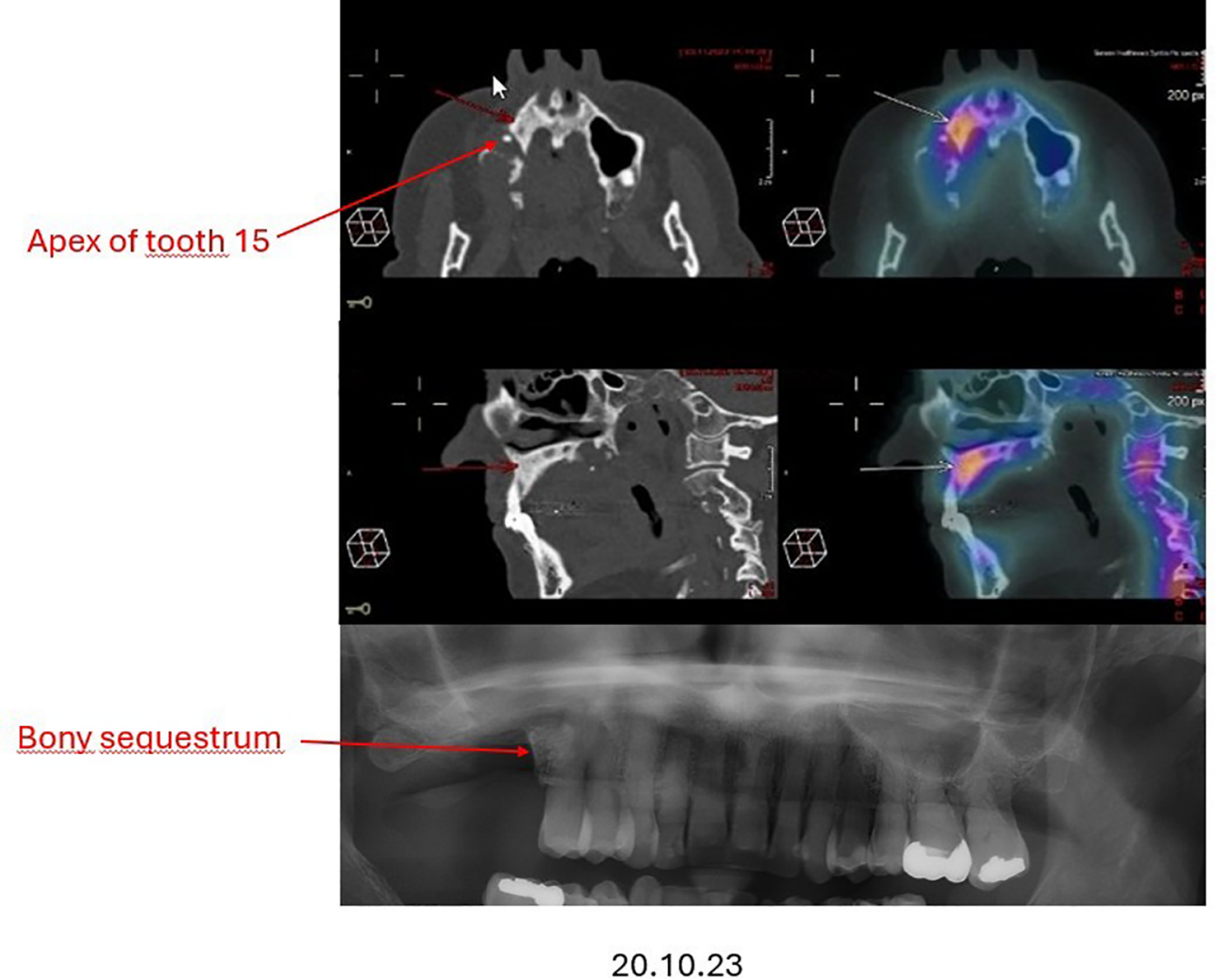
Osteonecrosis of the jaw in PET-CT and OPG.

Concurrently, following the extraction of the tooth 16 in July 2022, the patient reported persistent jaw pain and delayed healing with persistent bone exposure around tooth 15, raising suspicion of stage II osteonecrosis of the jaw (ONJ) (**Figure 2**). Management of this complication was delayed.

**Figure 3:**
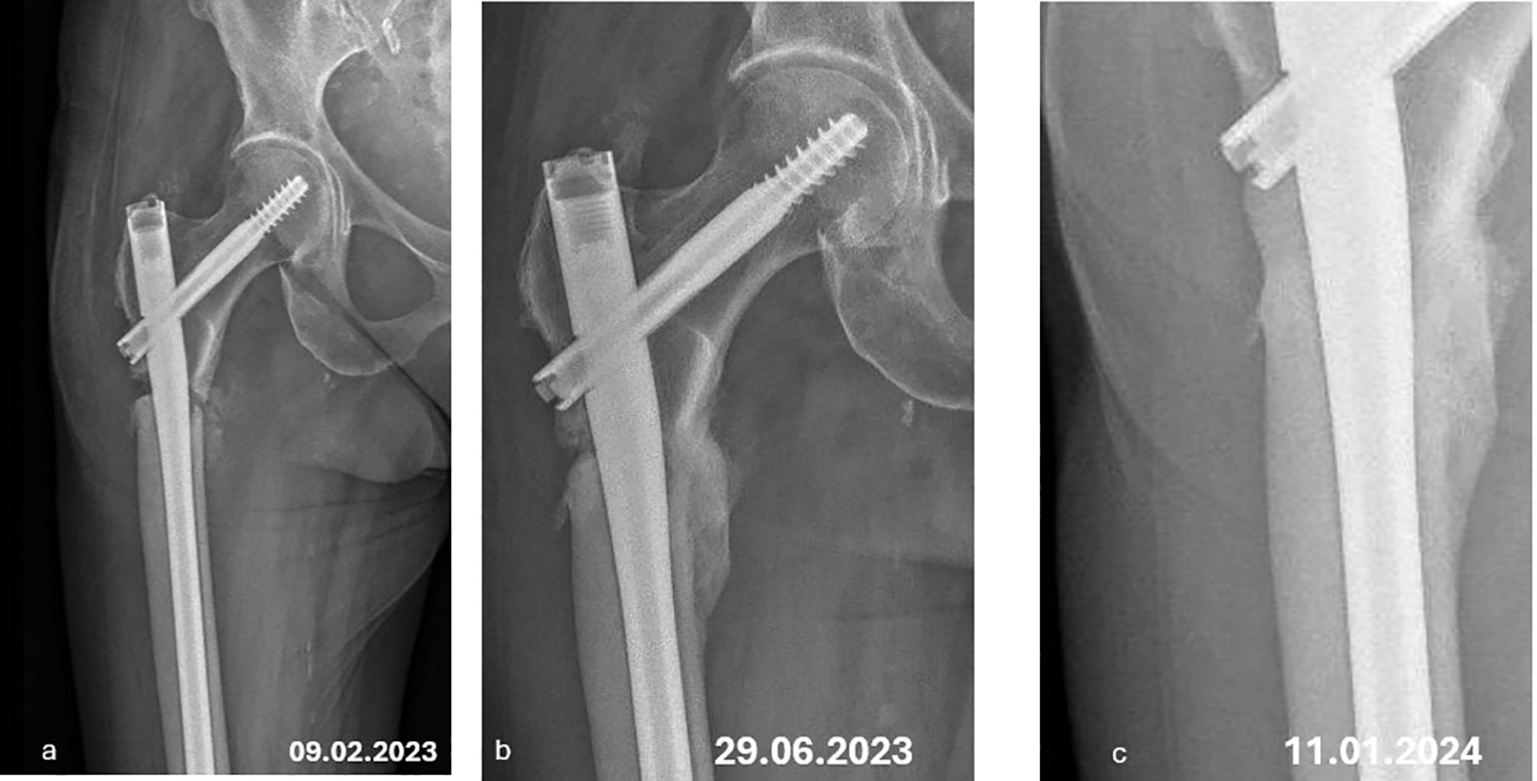
**(a)** atypical femur fracture with intramedullary nailing. **(b)** complete healing of the atypical femur fracture with bone callus. **(c)** hypertrophic callus at the fracture site.

Due to increasing femoral pain, an orthopedic evaluation was planned to consider prophylactic nailing. However, in December 2022, the patient suffered a fall from standing height, resulting in a complete femoral fracture requiring surgical fixation (**Figure 3a**).

Given the co-occurrence of AFF and suspected ONJ it was considered to discontinue denosumab. Instead, to prevent rebound bone loss and the risk of spontaneous vertebral fractures known to occur after abrupt denosumab withdrawal, a modified dosing strategy was implemented: denosumab was resumed at a reduced dose of 15 mg, administered every 3.5 months starting February 2023. The patient received in total 4 reduced doses of denosumab between February 2023 and April 2024. During this period, serum cross-linked C-terminal telopeptide of type I collagen (sCTX), a marker of bone resorption, were within the premenopausal range values (<530 ng/L; normal range 137-643) when dosed before the next denosumab injection (**Table 1**).

**Table 1:** Denosumab dose administration according to the sCTX monitoring.

Date	Denosumab dose(mg)	sCTX values (ng/L)
17.10.2022	120	49
Diagnosis of AFF		
31.01.2023	15	530
03.05.2023	15	317
AFF completion and surgery		
16.08.2023	15	351
12.07.2024	15	265
Spotaneous jawbone fragment loss		
03.04.2025	15	626
08.05.2025	–	834

sCTX, serum cross-linked C-terminal telopeptide of type I collagen; AFF, atypical femur fracture.

By June 2023, radiographic imaging showed complete healing of the femoral fracture (**Figure 3b**). Because of ONJ persistence by the end 2023 (bone exposure), surgical treatment was planned for January 2024. In December 2023, the patient reported the spontaneous loss of tooth 15 associated with a bone fragment from the jaw. By January 2024, a maxillofacial surgeon confirmed complete mucosal and bone healing despite no specific treatment.

As of the latest follow-up, the patient continues on low-dose denosumab (15 mg every 3 to 7 months, adjusted to maintain sCTX on the premenopausal range), with no recurrence of ONJ and progressive remodeling of the AFF site. The most recent radiographic control, on February 2025, (**Figure 3c**) shows a hypertrophic callus at the fracture site.

## Discussion

AFF and ONJ are rare but serious complications associated with long-term use of antiresorptive therapies, particularly bisphosphonates and denosumab.

The incidence of AFF under monthly denosumab (120 mg) therapy or after its discontinuation ranges between 0.4% and 1.8% versus 7 per 10,000 patient-years in osteoporosis patients (1). In a retrospective series, Takahashi et al. reported AFFs in 5 out of 277 cancer patients (1.8%) treated with an oncologic dose of denosumab, with long-term exposure (>3.5 years) and prior treatment with zoledronic acid identified as significant risk factors (2).

The incidence of ONJ is estimated to range from 0.001% to 0.01% in patients with osteoporosis (3). According to Everts-Graber et al., denosumab use is associated with a significantly increased risk of medication-related osteonecrosis of the jaw compared with zoledronic acid in this population (4). Its incidence is substantially greater, ranging from 0.7% to 15%, among oncology patients, who receive higher doses of denosumab at more frequent intervals (3, 5).

While both AFF and ONJ are individually rare, their coexistence in the same patient is even more uncommon. According to a review by Meyyur Aravamudan et al. combined cases are exceedingly infrequent and present unique management challenges (6).

Several guidelines have recommended discontinuation of antiresorptives in the event of either ONJ or AFF (3, 7). Bisphosphonates, while a bridging strategy for managing the post-denosumab rebound, are discouraged in this context, particularly due to their inability to counteract the rebound effect unless given in high doses, which may pose additional risks for ONJ and AFF. There is limited data on this rebound effect in oncology patients (8). This uncertainty further complicates treatment decisions following denosumab-associated adverse events.

We did not find any published data on the continuation of denosumab in ONJ. A systematic review of published cases of AFF under denosumab (7) suggested that continuing denosumab increased the risk of non-union even after surgery, as well as the risk of contralateral AFF, supporting denosumab discontinuation in this situation. However, some cases evolved positively under denosumab. For example, Peak et al. reported complete bone union after initiating denosumab 60 mg in a patient with bisphosphonate-related AFF, with full weight-bearing restored three months postoperatively (7). In this case, denosumab was prescribed for the management of low BMD and due to the inability to continue bisphosphonate therapy.

To date, no reports have described the use of low-dose denosumab following AFF or ONJ associated to either osteoporotic (60 mg every 6 months) or oncologic (120 mg every 1–3 months) denosumab dosing regimens. In the dose-finding clinical trial, withdrawal from 30 mg denosumab remained associated with a rebound in bone turnover markers (9). This led us to explore an even lower dose of 15 mg denosumab as a bridging therapy in our patient, aiming to minimize the rebound effect while reducing adverse event risk. Unexpectedly, this low-dose regimen was associated not only with accelerated healing of the AFF, but also with complete spontaneous resolution of ONJ, in the absence of any treatment (neither antibiotic nor surgery).

Several mechanisms may explain these observations: firstly, partial inhibition of RANKL at lower denosumab doses may prevent excessive suppression of bone remodeling while avoiding overactivation of osteoclast precursor cells. This is suggested by the sCTX levels at the premenopausal range before each next injection. Partial RANKL suppression might also prevent fusion of osteomorphs, a process implicated in pathologic bone remodeling in animal models (10).

Another plausible hypothesis is that low-dose denosumab permits local RANKL release at the fracture site. As shown in prior studies, microcracks lead to osteocyte apoptosis, which in turn stimulates the release of local signals (including RANKL), activating both osteoblasts and osteoclasts to initiate bone repair (11).

To our knowledge, this is the first reported case describing concurrent resolution of AFF and ONJ under continued low-dose denosumab therapy. While this favorable outcome is encouraging, it must be interpreted with caution given the inherent limitations of a single-case report, which precludes any generalization of efficacy or safety. Prospective studies and larger case series are clearly needed to better define the role of low-dose denosumab in patients with severe skeletal-related complications.

## Data Availability

The original contributions presented in the study are included in the article/supplementary material. Further inquiries can be directed to the corresponding author.
